# Acupuncture in ischemic stroke: from local neuromodulation to metabolic reprogramming

**DOI:** 10.3389/fneur.2026.1860817

**Published:** 2026-06-12

**Authors:** Shuhui Kan, Pengcheng Li, Lina Lu, Chenshu Zhang

**Affiliations:** 1 The Tenth Hanan Acupuncture Ward, The Second Affiliated Hospital of Heilongjiang University of Chinese Medicine, Harbin, Heilongjiang, China; 2 Department of Neurosurgery II, Heilongjiang Third Hospital, Harbin, Heilongjiang, China; 3 The Fourth Hanan Neurological Rehabilitation Ward, The Second Affiliated Hospital of Heilongjiang University of Chinese Medicine, Harbin, Heilongjiang, China

**Keywords:** acupuncture, ischemic stroke, metabolic reprogramming, neurovascular unit, precision rehabilitation

## Abstract

Ischemic stroke remains a leading cause of death and long-term disability worldwide, and its management extends far beyond hyperacute reperfusion to include rehabilitation and secondary prevention. Acupuncture has long been used as an adjunctive intervention in stroke rehabilitation, yet its clinical role remains unsettled because benefit signals vary across outcomes, study designs, and control conditions. In recent years, a more informative framework has emerged: ischemic stroke is not only a focal vascular event but also an evolving metabolic crisis involving mitochondrial dysfunction, altered glucose utilization, lactate imbalance, lipid peroxidation, ferroptosis, and systemic metabolic dysregulation. Against this background, the present narrative review examines acupuncture in ischemic stroke through the lens of metabolic reprogramming. Current clinical evidence suggests that acupuncture may show more consistent signals in selected domains such as dysphagia, depressive symptoms, and possibly cognitive impairment than in global disability as a whole. Mechanistic studies increasingly indicate that acupuncture may influence mitochondrial bioenergetics, lactate signaling, ferroptosis-related lipid injury, and microbiota-derived metabolic pathways, although most evidence remains preclinical. We further argue that post-stroke phenotypes differ in their metabolic sensitivity and that this heterogeneity may partly explain the persistent inconsistency of the field. Future research should move beyond undifferentiated efficacy claims and adopt biomarker-anchored, phenotype-specific, and rigorously controlled trial designs. A metabolism-centered framework may therefore help generate testable hypotheses for clarifying the potential adjunctive role of acupuncture in ischemic stroke recovery.

## 1 Introduction

Ischemic stroke remains one of the leading causes of death and long-term disability worldwide. In 2021, the global burden of ischemic stroke was still substantial, with an estimated 69.9 million prevalent cases, an age-standardized incidence rate of 92.4 per 100,000, and an age-standardized disability-adjusted life-year rate of 837.4 per 100,000 (1). At the broader stroke level, the burden continues to rise in absolute terms despite progress in prevention and acute care, and the annual global direct and indirect costs of stroke have been estimated to exceed US$891 billion, with macroeconomic losses reaching 1.66% of global GDP in 2019 (2, 3).

These figures make it clear that post-stroke management cannot be framed solely as a problem of hyperacute reperfusion. Effective rehabilitation and secondary prevention remain central to reducing disability, dependence, recurrence, and long-term societal cost after stroke (2). Against this background, acupuncture has remained one of the most widely used adjunctive interventions in stroke rehabilitation, particularly in East Asia, because it is relatively accessible, can be integrated with conventional therapy, and is increasingly investigated in randomized and mechanistic studies (4, 5). Recent meta-analyses suggest that acupuncture may improve neurological deficits, global disability, activities of daily living, and some functional outcomes after ischemic stroke, but the overall certainty of evidence is still mostly low to moderate (4, 5).

However, the clinical position of acupuncture in stroke remains unsettled. A sham-controlled meta-analysis published in 2025 reported that acupuncture was associated with improved neurological function, quality of life, and depressive symptoms, but not with a clear improvement in activities of daily living, and explicitly highlighted unresolved issues involving needling depth, acupuncture type, manual manipulation, de qi, and sham-control methodology (6). This uncertainty is mirrored at the guideline level. An evidence-based evaluation of stroke rehabilitation guidelines found relatively consistent support for acupuncture in post-stroke dysphagia, whereas recommendations for upper-extremity motor dysfunction and cognitive impairment remained controversial, conditional, or insufficiently developed (7).

One reason for this inconsistency may be that the field has too often asked whether acupuncture is beneficial after stroke, without first clarifying which biological dimension of stroke it is targeting. Ischemic stroke is not only a focal vascular occlusion but also an evolving metabolic crisis, in which bioenergetic failure, oxidative stress, lipid disturbance, and brain–body metabolic crosstalk shape both tissue injury and subsequent recovery (8). Consistent with this view, metabolomics studies have identified broad disruptions in amino-acid, lipid, and related metabolic pathways in ischemic stroke, supporting the use of metabolic profiling for mechanistic stratification, biomarker discovery, and outcome prediction (9). This metabolic lens is clinically relevant because cardiometabolic disease is tightly interwoven with stroke biology: diabetes is associated with worse acute outcomes, poorer post-stroke recovery, and greater recurrence risk (10), while metabolic syndrome is associated with increased stroke recurrence and all-cause mortality (11).

**Figure 1:**
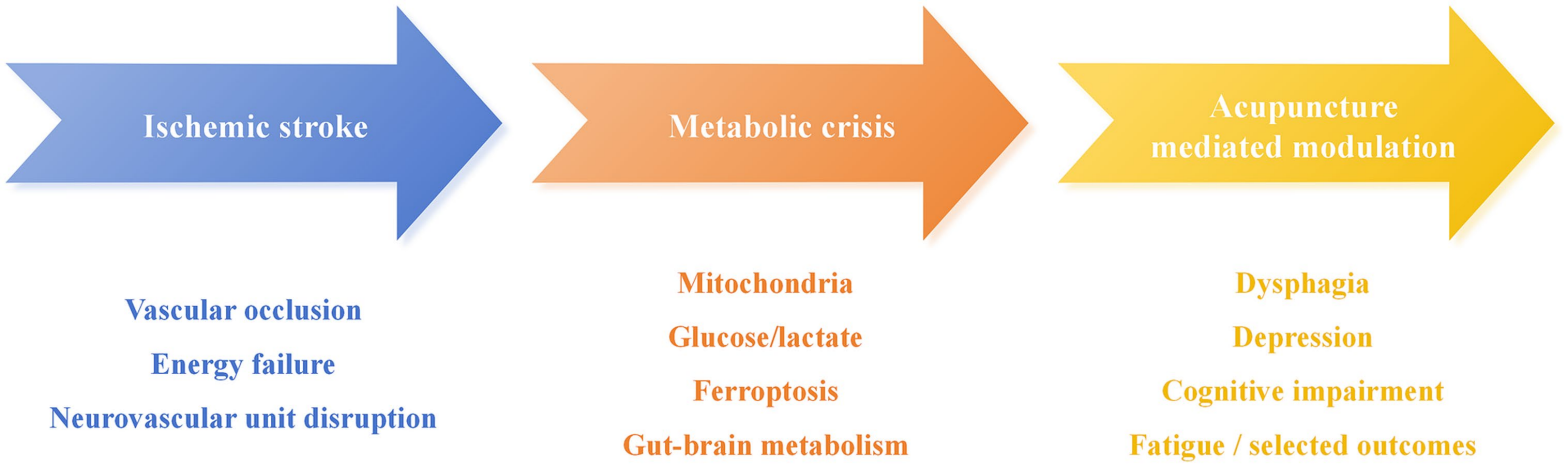
Conceptual framework linking ischemic stroke, metabolic crisis, and acupuncture-mediated phenotype-specific recovery.

Importantly, the emerging mechanistic literature on acupuncture increasingly converges on this metabolic axis. Recent reviews emphasize that acupuncture-related neuroprotection in ischemic stroke is closely linked to mitochondrial integrity, oxidative balance, mitochondrial membrane potential, and mitochondrial quality control (12). Experimental work in middle cerebral artery occlusion models has reported that acupuncture may increase ATP levels and mitochondrial complex I activity, reduce oxidative stress, and facilitate AMPK/PGC-1α-mediated mitochondrial biogenesis (13). These observations suggest that the more informative question is not simply whether acupuncture works after ischemic stroke, but whether metabolism-related pathways may help explain selected clinical benefit signals after ischemic stroke. On this basis, the present narrative review examines acupuncture in ischemic stroke through the framework of metabolic reprogramming, with a focus on how this perspective may help reconcile conflicting clinical findings, refine patient selection, and identify more plausible translational endpoints for future studies (Figure 1).

This article was designed as a narrative review rather than a systematic review or meta-analysis. To improve transparency, we conducted a targeted PubMed/MEDLINE search up to March 1, 2026, using terms related to ischemic stroke, acupuncture, electroacupuncture, stroke rehabilitation, metabolic reprogramming, mitochondria, lactate, lactylation, ferroptosis, gut microbiota, metabolomics, insulin resistance, and secondary prevention. We prioritized randomized trials, sham-controlled studies, systematic reviews, meta-analyses, clinical guidelines, mechanistic studies, and translational biomarker research directly relevant to ischemic stroke recovery or post-stroke metabolic regulation. Additional studies were identified from reference lists of key articles. Because this was a narrative review, formal risk-of-bias assessment, PRISMA-based screening, and quantitative synthesis were not performed. Evidence was summarized thematically, and the metabolism-centered framework proposed here should therefore be regarded as a translational hypothesis rather than established clinical proof.

## 2 Why metabolism matters in stroke recovery

The biological cascade of ischemic stroke begins as an energetic catastrophe. Once cerebral blood flow is interrupted, oxygen and glucose delivery fall abruptly, ATP production collapses, ion gradients fail, and a chain of excitotoxicity, oxidative injury, membrane destabilization, and cellular dysfunction is rapidly initiated (14). This is why metabolism should not be treated as a secondary byproduct of ischemic injury, but as one of its earliest and most determinative layers. Consistent with this view, recent metabolomics-focused work has further emphasized that ischemic stroke involves coordinated pathway disturbances rather than isolated biochemical abnormalities (15, 16).

A metabolism-centered perspective is also necessary because stroke recovery cannot be explained by neuronal survival alone. The older neuron-centered model has increasingly been replaced by a broader neurovascular-unit framework, in which neurons, astrocytes, endothelial cells, and surrounding vascular elements function as a coupled biological system (17). Within this network, astrocyte-neuron metabolic crosstalk is especially important in the peri-infarct milieu, where astrocytes help buffer excitotoxic stress, redistribute metabolic substrates, and support neuronal adaptation when oxidative metabolism is impaired (18). In parallel, endothelial glucose transport is now recognized as another critical determinant of recovery, because maintenance of endothelial GLUT1 function helps sustain glucose transfer across the blood–brain barrier, preserve endothelial integrity, and support post-stroke prognosis (19). Together, these findings suggest that meaningful recovery depends not only on reperfusion and synaptic reorganization, but also on re-establishing coordinated metabolic function across the neurovascular unit.

This framework is clinically relevant because the injured brain is tightly coupled to systemic metabolic status. Stroke outcome is shaped not only by lesion characteristics and recanalization status, but also by the host’s glycometabolic background, vascular phenotype, and peripheral biological response (17). Clinically, this is illustrated by the consistent association between dysglycemia and adverse outcomes after acute ischemic stroke. A dose–response meta-analysis found that higher stress hyperglycemia ratio was associated with increased risks of poor functional outcome, mortality, intracerebral hemorrhage, neurological deficits, and recurrence (20). Likewise, among patients treated with intravenous thrombolysis, elevated blood glucose was associated with greater risks of symptomatic intracranial hemorrhage, poor 90-day functional outcome, and death (21). These data reinforce a key point for this review: metabolism is not merely a mechanistic curiosity in ischemic stroke, but a clinically visible determinant of recovery trajectory and treatment responsiveness.

Placing metabolism at the center of stroke recovery also provides a more coherent translational framework. Clinical metabolomics studies have shown that circulating metabolic signatures can contribute to risk stratification and prognosis, and one untargeted metabolomics study demonstrated that acute-stage metabolite profiles were associated with 3-month functional outcome after ischemic stroke (22). More broadly, metabolomics-based reviews suggest that ischemic stroke should be understood through integrated pathway disruption rather than through any single biomarker alone (15). This matters because many long-standing inconsistencies in adjunctive stroke therapies may reflect hidden biological heterogeneity. Patients with apparently similar clinical syndromes may differ substantially in mitochondrial resilience, substrate utilization, oxidative burden, endothelial transport capacity, and systemic metabolic stress. A metabolism-first framework is therefore attractive not because it replaces inflammation, apoptosis, or ferroptosis, but because it helps integrate them into a shared biological architecture of injury and recovery. For a review on acupuncture, this framing is particularly important, because it creates a plausible bridge between local neuromodulation and whole-body biological response after ischemic stroke (14–16, 22).

## 3 What does the clinical evidence really show?

The clinical literature on acupuncture in stroke has evolved less as a linear progression toward acceptance than as a repeated oscillation between enthusiasm and skepticism. Early rigorous synthesis was notably cautious. In 2001, Park et al. (23) concluded that there was no compelling evidence that acupuncture improved stroke rehabilitation when attention was restricted to the more rigorous randomized trials. More than a decade later, an overview of systematic reviews refined rather than fully reversed that position. It suggested that acupuncture might improve post-stroke neurological impairment and dysfunction, particularly dysphagia-related outcomes, but still did not clearly reduce death, dependency, or broader motor dysfunction (24). These earlier syntheses already exposed the central problem that continues to define the field today: benefit signals are repeatedly observed, yet the most policy-relevant endpoints remain the hardest to stabilize.

In more recent evidence syntheses focused specifically on ischemic stroke, the overall tone has become more positive. A 2024 meta-analysis with trial sequential analysis reported significant improvements in NIHSS, mRS, and BI or MBI, although the certainty of evidence ranged from moderate to low (4). Another 2024 systematic review of acute ischemic stroke similarly suggested that acupuncture may improve neurological deficit scores, activities of daily living, motor outcomes, and prognosis, but again emphasized that the certainty of evidence was low (5). However, the most clinically revealing update may be the 2025 sham-controlled meta-analysis. When acupuncture was compared with sham or placebo acupuncture rather than routine care alone, benefits remained detectable for neurological function, stroke-specific quality of life, and depressive symptoms, but not for Barthel Index-defined daily living ability (6). This pattern suggests that part of the apparent efficacy seen in conventional meta-analyses may be magnified by co-intervention effects, expectancy, or insufficiently inert control conditions, whereas some outcome domains remain detectable even under more rigorous comparison.

The endpoint-specific literature is even more informative. A 2025 evaluation of stroke rehabilitation guidelines found the highest degree of agreement for recommending acupuncture in post-stroke dysphagia, whereas recommendations for upper-extremity motor dysfunction and cognitive impairment still required further development (7). This hierarchy is broadly consistent with recent symptom-specific syntheses. A 2025 meta-analysis with trial sequential analysis reported positive effects of acupuncture on multiple swallowing-related outcomes and swallowing-related quality of life in post-stroke dysphagia (25). A 2024 meta-analysis on post-stroke cognitive impairment found improvements in MoCA and MMSE, but also explicitly stated that long-term efficacy and any impact on recurrence remained undetermined (26). For post-stroke spasticity, a 2024 overview judged acupuncture promising, yet also found that most included reviews were of low or critically low methodological quality (27). In other words, the evidence base appears strongest for selected symptom complexes such as dysphagia, more fragile for cognition and spasticity, and still difficult to generalize to global disability recovery.

From a translational perspective, the key question is therefore not simply whether acupuncture works after stroke, but in which patients, at which stage, and for which endpoint. The literature remains markedly heterogeneous in treatment timing, acupuncture modality, dose, sham design, co-interventions, and outcome measurement (6, 23, 24, 27). This heterogeneity helps explain why the field can simultaneously produce positive pooled estimates and persistent guideline caution. It also suggests that future trials should move away from undifferentiated efficacy claims and toward more explicitly stratified models of response, rather than treating all stroke survivors as a single therapeutic population.

## 4 Acupuncture and post-stroke metabolic reprogramming

If acupuncture has effects beyond symptom-level modulation in ischemic stroke, metabolism may provide a plausible framework for interpreting those effects. Cerebral ischemia is fundamentally a disorder of interrupted substrate delivery and maladaptive metabolic adaptation, with downstream consequences involving mitochondrial dysfunction, altered glucose handling, lipid peroxidation, oxidative stress, and overlapping cell-death programs (14–16, 22). Notably, the recent acupuncture-mechanism literature has started to converge on the same terrain. Rather than describing acupuncture only as a generic anti-inflammatory or neuroprotective intervention, newer studies suggest that it may influence mitochondrial bioenergetics, lactate dynamics, ferroptosis, and gut-derived metabolic signaling (12, 28, 29).

### 4.1 Mitochondrial rescue and bioenergetic restoration

Among the available mechanistic signals, the mitochondrial axis is the most coherent. A 2024 review focused on neuronal mitochondria summarized acupuncture-associated effects on mitochondrial structural integrity, oxidative stress, calcium overload, membrane potential, and mitochondrial quality control in ischemic stroke models, suggesting that mitochondrial rescue may sit near the center of acupuncture’s experimental neuroprotective profile (12). Experimental work supports this interpretation. Guo and Lu reported that acupuncture in MCAO rats increased ATP levels and mitochondrial respiratory chain complex I activity, reduced oxidative stress-related signaling, and activated the AMPK/PGC-1α axis, supporting a mitochondrial biogenesis-centered mechanism (13). Wang et al. (28) further showed that electroacupuncture ameliorated neuronal injury by promoting Pink1/Parkin-mediated mitophagy clearance and reducing nitro-oxidative stress, providing additional support for a mitochondria-centered mechanism. Lou et al. (29) likewise found that acupuncture after ischemic injury increased oxidative phosphorylation complex proteins, ATP concentration, and mitochondrial membrane potential while improving redox balance, again linking acupuncture to restoration of energy production rather than only downstream injury suppression. Taken together, these data suggest that mitochondrial rescue is not a peripheral mechanism but one of the core metabolic nodes through which acupuncture may influence post-ischemic recovery. However, most evidence supporting this mitochondrial mechanism comes from animal models of cerebral ischemia–reperfusion injury. Whether acupuncture-induced changes in mitochondrial bioenergetics can be detected in human stroke survivors, and whether such changes mediate functional recovery, remains to be established.

### 4.2 Glucose handling, lactate flux, and lactylation

A second emerging theme is the regulation of glucose utilization and lactate biology. Earlier experimental work showed that electroacupuncture up-regulated astrocytic monocarboxylate transporter 1 in MCAO rats, enhanced lactate-related energy metabolism in the ischemic cortex, and was associated with better neurological recovery, supporting the idea that acupuncture may influence astrocyte-neuron substrate shuttling after stroke (30). More recent work has added a new layer by implicating lactylation. Pan et al. (31) reported that electroacupuncture pretreatment reduced ischemic brain damage while inhibiting lactate production and its derived protein lactylation, suggesting that acupuncture may also restrain maladaptive consequences of excessive glycolytic stress. This makes the broader lactate literature highly relevant, because lactate in ischemic stroke is no longer viewed as a simple toxic by-product; it is increasingly understood as both an energy substrate and an immunometabolic signal, with lactylation adding an additional regulatory dimension (32). A reasonable synthesis of these findings is not that acupuncture uniformly suppresses glycolysis, but that it may help rebalance lactate production, transport, and signaling during post-ischemic recovery. At present, the lactate-lactylation axis should therefore be interpreted as a mechanistically attractive but clinically unvalidated pathway. Future human studies will need serial metabolic profiling to determine whether acupuncture modifies lactate-related signaling in parallel with recovery outcomes.

### 4.3 Lipid peroxidation, iron handling, and ferroptosis

The third major metabolic axis is ferroptosis, which is especially attractive because it links iron dysregulation, lipid peroxidation, oxidative stress, and mitochondrial injury into a single pathophysiological program. More general stroke literature has already established ferroptosis as one of the relevant cell-death mechanisms in cerebral ischemia–reperfusion injury (22). Within acupuncture research, Wang et al. (33) reported that electroacupuncture improved neurological deficits, reduced infarct volume, alleviated mitochondrial morphological injury, decreased ACSL4 and TFR1 expression, and increased GPX4 levels in MCAO/R rats, supporting a ferroptosis-related mechanism. Zhu et al. (34) further showed that electroacupuncture reduced neuronal damage and infarct burden while increasing Nrf2, SLC7A11, and GPX4 signaling, consistent with suppression of ferroptotic injury through an antioxidant defense axis. Other recent studies extended this direction by linking electroacupuncture-mediated suppression of oxidative stress and ferroptosis to the mTOR/SREBP1 pathway (35), while a 2025 Brain Research study suggested that AMPK activation itself may mediate the anti-ferroptotic effect of acupuncture in cerebral ischemia–reperfusion models (36). The broader implication is important: acupuncture may not be acting on a single downstream injury pathway, but on a metabolically upstream network in which iron handling, membrane lipid vulnerability, and redox control converge. These findings support ferroptosis as a plausible experimental target of acupuncture, but direct evidence in human post-stroke recovery is still lacking. Clinical validation would require peripheral or imaging-compatible biomarkers of lipid peroxidation, iron handling, or antioxidant response.

### 4.4 Gut microbiota-derived metabolites and the brain-gut axis

The gut-metabolic axis is the most exploratory but also one of the most interesting directions in this field. In a rat MCAO/R study, Ke et al. (37) found that electroacupuncture increased peripheral short-chain fatty acids and linked these changes to improvements in neurological function, motor function, and infarct-related outcomes, supporting a microbiota-metabolite component to acupuncture’s effects after stroke. A more recent study by Chen et al. (38) reported that electroacupuncture reprogrammed gut microbiota composition after ischemic stroke and conferred cerebral protection together with an enhanced regulatory T-cell response, strengthening a microbiome-gut-brain interpretation. In addition, a combined therapy study using acupuncture plus NaoMaiTong identified coordinated changes in gut microbiota and plasma metabolites, suggesting that intestinal microecology and systemic metabolism may jointly participate in recovery, although the combination design makes it difficult to isolate acupuncture-specific effects (39). At present, this part of the literature should still be interpreted cautiously. It is mechanistically promising, but it remains dominated by preclinical work and is still some distance from human studies capable of validating microbiota-derived metabolic signatures as clinically useful mediators of acupuncture response. Importantly, microbiota changes observed in animal studies cannot be assumed to represent causal mediators of clinical recovery without intervention studies that link microbial metabolites, immune changes, and functional outcomes in the same patients.

Overall, the mechanistic literature supports a shift in how acupuncture is conceptualized in ischemic stroke. The most useful model is no longer one in which acupuncture is simply said to reduce inflammation or protect neurons. A more integrated interpretation is that acupuncture may influence post-ischemic recovery by reorganizing metabolic stress across several connected layers, including mitochondrial biogenesis, lactate handling, ferroptosis-related lipid injury, and microbiota-derived metabolite signaling. At the same time, most of these data come from animal models, many use pretreatment or tightly controlled reperfusion paradigms, and few directly bridge mechanistic markers with clinically meaningful human endpoints. Metabolic reprogramming is therefore best viewed as a hypothesis-generating framework and a potential translational direction, rather than as a proven clinical mechanism (12, 28–39).

## 5 Beyond the brain: systemic metabolic effects of acupuncture after stroke

From the standpoint of secondary prevention, the relevance of acupuncture after ischemic stroke cannot be judged only by its effects on neurological symptoms. The 2021 AHA/ASA guideline places control of vascular risk factors at the center of post-stroke management, including blood pressure, diabetes, lipids, obesity, diet, and physical activity (40). Among these, blood pressure remains the most important modifiable determinant of recurrent stroke. A contemporary review emphasized that blood pressure lowering reduces recurrent stroke risk by roughly 25 to 30%, and that a target below 130/80 mmHg is generally supported for many patients after stroke (41). A more recent meta-analysis further showed that intensive blood pressure control reduced recurrent stroke and major cardiovascular events in patients with prior stroke, although hypotension-related symptoms increased modestly (42). Glycometabolic risk is similarly important. A 2024 systematic review found that prediabetes and insulin resistance were both associated with recurrent vascular events after ischemic stroke or transient ischemic attack, and the IRIS trial showed that treating insulin-resistant, non-diabetic patients with pioglitazone reduced stroke or myocardial infarction and lowered incident diabetes, albeit at the cost of weight gain, edema, and fractures (43, 44). Together, these data make a simple point: if acupuncture is to have relevance beyond symptomatic rehabilitation, its potential effects on systemic metabolic risk domains should be evaluated cautiously in the context of standard secondary prevention.

Against this background, the most plausible extra-cerebral signal for acupuncture lies in insulin resistance and broader metabolic syndrome. A systematic review and meta-analysis concluded that acupuncture may improve HOMA-IR, insulin sensitivity index, fasting blood glucose, 2-h glucose, and fasting insulin, with fewer adverse events than comparator treatments in insulin-resistant populations (45). At a broader syndrome level, a meta-analysis of acupuncture for metabolic syndrome found reductions in waist circumference and body mass index, alongside improvements in fasting blood glucose and several hyperlipidemia-related indices (46). These findings are relevant to stroke because they suggest that acupuncture may exert effects not only on the injured brain but also on the peripheral metabolic milieu that shapes vascular recurrence risk and recovery capacity. At the same time, the translational gap should not be ignored. Most of this evidence comes from non-stroke metabolic populations, uses relatively short follow-up, and does not demonstrate that metabolic improvement through acupuncture translates into fewer recurrent cerebrovascular events after stroke.

The evidence for adiposity-related benefit is somewhat more mature, but still not definitive. An umbrella review published in 2025 found that the strongest evidence supported acupuncture combined with lifestyle interventions, compared with lifestyle interventions alone, for improving body mass index and body weight in obesity (47). This is clinically attractive because excess adiposity often coexists with hypertension, insulin resistance, dyslipidemia, and chronic inflammation, all of which are relevant to long-term cerebrovascular risk. Even so, the step from modest weight reduction to improved post-stroke prognosis remains unproven. For a stroke-focused review, the key point is therefore not that acupuncture has already been shown to reduce recurrent stroke through weight control, but that it may influence one component of the broader cardiometabolic profile that underlies recurrence and incomplete recovery (40, 47).

Blood pressure is the domain in which the clinical promise of acupuncture is most directly relevant to stroke, but also where the evidence is most cautionary. A Cochrane review concluded that there was no evidence for the sustained blood-pressure-lowering effect of acupuncture that would be required for chronic hypertension management, and that the apparent larger effects in non-sham-controlled studies were most likely explained by bias rather than true efficacy (48). In stroke patients specifically, a systematic review on acupuncture for blood pressure regulation reported that some small trials observed lower blood pressure after acupuncture, including in cerebral infarction cohorts, but the evidence base was limited and methodologically weak (49). This creates an important tension in interpretation. Because intensive blood pressure control clearly matters for secondary stroke prevention, even a modest durable antihypertensive effect from acupuncture would be clinically meaningful. However, the current literature does not yet provide robust proof that acupuncture can deliver such an effect in a way that is reliable enough to be incorporated as an evidence-based vascular risk-modifying strategy after stroke (41, 42, 48, 49).

Overall, the extra-cerebral case for acupuncture in ischemic stroke is plausible but not yet conclusive. The available evidence supports the idea that acupuncture can influence systemic metabolic domains including insulin resistance, body composition, fasting glucose, and some lipid-related indices, while the evidence for durable blood pressure control remains substantially weaker (45–49). What is still missing is the decisive translational step: trials showing that these peripheral metabolic changes mediate lower recurrence, better long-term function, or more effective secondary prevention after stroke. For now, acupuncture is best viewed not as an established systemic vascular risk-modifying therapy, but as a potentially valuable adjunct whose metabolic effects may help explain benefit in selected stroke phenotypes and deserve more targeted prospective study (40–49).

## 6 From mechanisms to phenotypes: which post-stroke syndromes are most likely to be metabolism-sensitive?

One practical implication of the preceding sections is that not all post-stroke phenotypes are equally informative for testing a metabolism-centered model of acupuncture. Some outcomes are dominated by network disconnection, corticospinal tract injury, or lesion topology, whereas others appear to sit closer to persistent metabolic stress, neuroinflammation, and systemic biological maladaptation. This distinction matters because the field has often treated stroke recovery as a single therapeutic domain, even though different post-stroke syndromes are likely to have different biological drivers and different probabilities of responding to adjunctive therapies. In motor recovery, for example, recent reviews emphasize that prognostic performance is improved mainly by biomarkers of corticospinal tract structure and function, neurophysiology, and systems-level reserve rather than by any single metabolic marker (50, 51). This does not make metabolism irrelevant to motor recovery, but it suggests that pure motor deficit may be a less specific test case for a metabolic-reprogramming hypothesis than other post-stroke syndromes.

By contrast, post-stroke cognitive impairment appears to be a particularly strong candidate for a metabolism-sensitive phenotype. The 2023 AHA/ASA scientific statement emphasized that cognitive impairment is common after stroke, especially in the first year, and that up to one-third of stroke survivors develop dementia within 5 years, often in the setting of preexisting microvascular and neurodegenerative changes (52). This framing is important because cognition after stroke is influenced not only by focal lesion burden but also by whole-brain vulnerability, vascular reserve, and chronic cardiometabolic background. A 2024 meta-analysis further showed that post-stroke cognitive impairment was associated with significantly higher risks of recurrent stroke and all-cause mortality, suggesting that cognitive dysfunction is not merely a parallel symptom but a marker of broader biological instability after stroke (53). In that context, the modestly positive signal seen for acupuncture in post-stroke cognitive impairment becomes more interesting: the 2024 meta-analysis cited earlier found improvements in MoCA and MMSE, even though long-term efficacy and effects on recurrence remained uncertain (26). Taken together, these findings suggest that cognition may be one of the most relevant phenotypes in which a metabolism-centered model of acupuncture deserves serious testing.

Post-stroke depression is another phenotype that fits the metabolic and immunobiological framework particularly well. A widely cited updated review described post-stroke depression as common and associated with higher mortality, poorer recovery, greater cognitive deficits, and lower quality of life than stroke without depression (54). More importantly for the present review, the biological discussion of post-stroke depression increasingly overlaps with the pathways highlighted in metabolic reprogramming, including neuroinflammation, altered neurotransmitter regulation, stress signaling, and brain–body immune interaction. A 2025 meta-analysis reported that inflammatory cytokines differed between patients with and without post-stroke depression and concluded that such cytokines may serve as biomarkers that inform its pathophysiology (55). This helps explain why depression has remained one of the few domains in which acupuncture continues to show a signal even in sham-controlled synthesis (6). Among post-stroke syndromes, depression therefore stands out as a phenotype where the clinical literature and the mechanistic literature are unusually well aligned.

Post-stroke fatigue may be an even more explicitly metabolism-linked phenotype, although it is methodologically less mature. A 2023 review described post-stroke fatigue as an independent and highly prevalent symptom, affecting roughly 42 to 53% of stroke survivors, and noted that biological, physical, and psychological factors all contribute, with inflammation potentially playing a key role (56). An earlier review went further by framing fatigue and depression as components of post-stroke sickness behavior triggered by stroke-induced systemic inflammation (57). This line of thinking is highly compatible with a metabolism-first model, because fatigue is difficult to explain by focal lesion anatomy alone and more plausibly reflects persistent dysregulation across energy metabolism, inflammatory signaling, and organism-level adaptation. Clinically, this phenotype also matters because a 2024 prospective longitudinal study found that early clinically significant fatigue predicted a poorer trajectory of functional independence during recovery (58). The difficulty, however, is that fatigue remains under-measured and under-standardized in stroke trials, including acupuncture trials. For that reason, fatigue may be one of the most biologically attractive but operationally underused phenotypes for future metabolism-oriented studies.

A broader lesson emerges from comparing these phenotypes. Dysphagia may be one of the most consistently positive endpoints for acupuncture at the guideline and meta-analysis level (7, 25), but it is not yet the clearest example of a metabolism-sensitive phenotype. Motor recovery is clinically central, but its prognosis is still strongly dominated by structural and systems-neuroscience markers (50, 51). In contrast, cognition, depression, and fatigue appear more deeply embedded in the intersection of vascular burden, inflammatory signaling, energy failure, and systemic biological reserve. These domains may therefore provide a more coherent testing ground for the hypothesis that acupuncture exerts benefit in ischemic stroke by reshaping metabolism rather than merely by producing nonspecific symptomatic effects. Taken together, these observations suggest that post-stroke phenotypes differ in both clinical signal and metabolic sensitivity, as summarized in Table 1. A useful next step for the field would be to stop asking whether acupuncture works for “stroke” in general, and instead ask in which biologically defined post-stroke phenotypes it is most likely to work, when given at the right stage and measured with the right endpoints.

**Table 1:** Phenotype-specific summary of the clinical signal and metabolic relevance of acupuncture in ischemic stroke.

Post-stroke phenotype/domain	Clinical signal for acupuncture	Metabolic relevance	Implication for future studies	References
Global disability/activities of daily living	Overall findings are inconsistent. Conventional meta-analyses suggest possible benefit, but sham-controlled evidence does not support a stable improvement in ADL.	Low to moderate; this is a broad composite outcome influenced by many non-metabolic factors.	Better used as a secondary endpoint than as the main test of a metabolism-centered hypothesis.	(4–6, 23, 24, 59)
Dysphagia	The most consistent positive clinical signal at both meta-analysis and guideline levels.	Moderate; clinically responsive, but not yet the clearest metabolism-sensitive phenotype.	Suitable for confirmatory clinical trials, although mechanistic metabolic endpoints still need strengthening.	(7, 24, 25)
Cognitive impairment	Positive signals have been reported, but long-term efficacy and effects on recurrence remain uncertain.	High; cognition is closely linked to vascular reserve, cardiometabolic burden, and whole-brain vulnerability.	One of the best targets for biomarker-anchored and metabolomics-informed studies.	(7, 26, 52, 53)
Depression	Relatively robust signal, including in sham-controlled analyses.	High; overlaps with inflammation, stress signaling, and brain–body immune-metabolic interaction.	A strong candidate for phenotype-specific trials with inflammatory and metabolic biomarkers.	(6, 54, 55)
Fatigue	Direct acupuncture evidence is limited, but the phenotype is biologically attractive.	High; fatigue likely reflects persistent dysregulation in energy metabolism and inflammatory signaling.	Promising exploratory target, especially for mechanism-oriented studies.	(56–58)
Motor dysfunction/spasticity	Some studies suggest benefit, but the overall evidence remains heterogeneous; for spasticity, methodological quality is often low.	Moderate; motor recovery is still heavily determined by structural and neurophysiological factors.	Should be studied with better stratification and more standardized outcome measures.	(5, 7, 24, 27, 50, 51, 62)
Systemic metabolic risk modification	Acupuncture may improve insulin resistance, glucose-related indices, and body composition, but durable blood pressure control and stroke recurrence reduction remain unproven.	High; systemic metabolic status is central to secondary prevention after stroke.	Relevant as an adjunctive translational direction, but not yet an established vascular risk-modifying strategy.	(40–49)

## 7 Why does the field remain inconsistent?

The persistence of inconsistency in acupuncture-stroke research is not accidental. It reflects a structural problem in trial design rather than a simple balance between “positive” and “negative” studies. Even in a relatively rigorous sham-controlled systematic review published in 2010, Kong et al. (59) found no significant benefit of acupuncture for functional recovery, with high heterogeneity across acute, subacute, and chronic-stage studies and substantial uncertainty around the adequacy of sham controls. Fifteen years later, the 2025 sham-controlled meta-analysis still reached only a partial and domain-specific conclusion, showing signals for neurological function, quality of life, and depressive symptoms, but not for activities of daily living, while again highlighting unresolved issues related to needling depth, acupuncture type, manual manipulation, de qi, and sham-control design (6). This continuity across time suggests that the central challenge in the field is not merely insufficient sample size, but instability in what is actually being compared, when it is being delivered, and which outcomes are being asked to respond.

A second source of inconsistency is temporal heterogeneity. Acupuncture is frequently discussed as if it were being applied to a single biological state called “stroke recovery,” yet acute, subacute, and chronic stroke represent very different therapeutic contexts. The Cochrane review on subacute and chronic stroke highlighted that these stages should be evaluated as a distinct clinical problem rather than merged indiscriminately with earlier phases (60). Similarly, the systematic review of acupuncture combined with rehabilitation in acute or subacute stroke concluded that any apparent benefit had to be interpreted against high risk of bias and a lack of consensus on the most appropriate acupuncture modality and treatment duration (61). When trials spanning different post-stroke stages, intervention schedules, and recovery windows are pooled together, apparent inconsistency may simply reflect biological non-equivalence rather than true contradiction.

A third problem is outcome heterogeneity. Stroke rehabilitation trials have long suffered from the use of too many non-harmonized endpoints, and acupuncture trials are no exception. A 2025 systematic review of upper-extremity outcome measures in post-stroke rehabilitation RCTs identified 112 different measures, with strong skew toward body function and activity domains and much less representation of participation-level outcomes (62). Under these conditions, two studies may both claim to test “motor recovery” while in reality measuring substantially different constructs. This becomes even more problematic in acupuncture research, where some trials prioritize NIHSS, others Fugl-Meyer scores, others Barthel Index, others symptom-specific scales, and follow-up timing is often inconsistent. The result is a literature that is statistically poolable but conceptually fragmented.

Reporting quality remains another major reason why conclusions are unstable. A 2014 evaluation of acupuncture RCTs for stroke rehabilitation found that contradictory findings were related in part to insufficient transparency and incomplete reporting, with multiple methodological details inadequately described (63). A 2017 assessment focusing specifically on post-stroke rehabilitation reached an even sharper conclusion: reporting quality was unsatisfactory, and crucial items such as trial design, outcomes, sample size, allocation concealment, blinding, flow charts, and intention-to-treat analysis were very poorly reported, while key acupuncture-intervention details such as number of needle insertions, responses sought, and needle type were also incompletely described (64). These are not minor editorial issues. When methodological and intervention details are underreported, the reader cannot determine whether two apparently similar acupuncture studies are truly comparable, nor whether a negative sham-controlled result reflects lack of efficacy, inadequate dosing, or an overly active control.

This is precisely why reporting standards matter so much in this field. The revised STRICTA recommendations were developed as an official extension of CONSORT to improve reporting of acupuncture rationale, needling details, treatment regimen, practitioner background, and control or comparator interventions (65). More recently, the SHARE guideline was proposed to improve the reporting of sham acupuncture in clinical trials, acknowledging that sham procedures themselves vary enough to distort interpretation if inadequately described (66). In principle, these tools should reduce ambiguity. In practice, however, many stroke-acupuncture trials either predate them or do not appear to have implemented them fully. As a result, uncertainty about dose, fidelity, and control adequacy continues to be built into the evidence base from the moment trials are published.

Finally, the field remains inconsistent because it has not yet fully entered a precision-rehabilitation paradigm. The Stroke Recovery and Rehabilitation Roundtable explicitly recommended the use of biomarkers to improve patient selection, stratification, and interpretation in stroke recovery trials, rather than relying on a one-size-fits-all model (67). More recent reviews of precision post-stroke rehabilitation have reinforced this direction by arguing for the integration of molecular, imaging, electrophysiological, and computational biomarkers to individualize prognosis and treatment response (68). This matters directly to acupuncture. If stroke recovery is biologically heterogeneous, then asking whether acupuncture works for “stroke” as a whole is probably the wrong question. A more informative approach would be to ask whether it works in specific phenotypes defined by stage, network reserve, metabolic profile, inflammatory burden, or symptom cluster. Until trials are designed around this logic, inconsistency will remain a predictable feature of the literature rather than an anomaly.

## 8 A translational roadmap for the next generation of studies

If the field is to move beyond repetitive meta-analyses of heterogeneous trials, the first change must be conceptual. Future studies should stop asking whether acupuncture works for “stroke” in the broadest sense and instead ask whether it works for biologically defined post-stroke phenotypes. The Stroke Recovery and Rehabilitation Roundtable has already argued that stroke recovery research needs agreed definitions, common language, and a framework that links biomarkers, measurement, and intervention development (67, 69). More recent precision-rehabilitation work has extended this logic by proposing integrated molecular, imaging, electrophysiological, and computational biomarkers for individualized recovery prediction (68). For acupuncture research, this means enrollment should be stratified not only by syndrome label or lesion side, but also by recovery stage, clinical phenotype, and where feasible, biomarker profile. A trial focused on post-stroke depression, cognitive impairment, or fatigue in metabolically vulnerable patients is more likely to generate biologically coherent results than a trial that pools all stroke survivors under a single umbrella diagnosis.

The second change must be methodological: future acupuncture trials should become biomarker-embedded rather than biomarker-adjacent. The 2025 Stroke recommendations for blood biospecimen research emphasized that stroke recovery studies have needs distinct from acute stroke and risk research, and specifically recommended longitudinal sampling, with a minimum of three time points unless the aim is purely predictive or genetic (70). This is highly relevant to acupuncture because any claim of “metabolic reprogramming” should be demonstrated dynamically rather than inferred from a single baseline or post-treatment snapshot. A recent multicenter prospective study offers a useful example of the type of design that could be integrated into next-generation trials: endostatin, GDF-10, and uPAR were sampled before rehabilitation and again during follow-up at 1, 3, and 6 months, and these markers showed associations with functional trajectories during recovery (71). In parallel, the emerging metabolomics literature continues to support the idea that stroke-related metabolic alterations may aid diagnosis, prognosis, and therapeutic interpretation (22, 72). The practical implication is straightforward: future acupuncture studies should prespecify serial blood collection, bank biospecimens prospectively, and test whether metabolic change tracks with clinical improvement over time rather than treating mechanistic analysis as an optional add-on.

The third change must address intervention fidelity and control design. Even a well-stratified biomarker-rich trial will remain difficult to interpret if acupuncture dose, practitioner behavior, sham procedures, and co-interventions are poorly specified. That is why STRICTA remains essential for reporting the rationale, needling details, treatment regimen, practitioner background, and control conditions (65). The newer SHARE guideline is equally important because sham acupuncture is not a single inert comparator; if sham procedures are incompletely described, apparent efficacy or lack of efficacy becomes hard to interpret (66). Encouragingly, some newer trial protocols are beginning to move in the right direction. A 2025 randomized sham-controlled protocol for upper-limb dysfunction after stroke used a principal-investigator-blinded design, central randomization, identical routine basic treatment in both groups, and surface electromyography as an auxiliary objective measure alongside conventional scales (73). Although this is only a protocol rather than a completed efficacy study, it illustrates the methodological direction the field should take: tighter control conditions, better blinding logic, and incorporation of objective physiological readouts rather than reliance on subjective scales alone.

The fourth change concerns outcome architecture. The field has already shown that post-stroke rehabilitation trials often use too many non-harmonized measures, with recent review work identifying 112 different upper-extremity outcomes across randomized trials (62). Future acupuncture studies should therefore adopt a more disciplined hierarchy of endpoints. Each trial should prespecify one primary clinical outcome that is phase-appropriate and phenotype-specific, a small number of secondary functional outcomes, and at least one objective biological or physiological outcome capable of testing mechanism. Just as importantly, studies should move from parallel reporting toward mediation testing. If acupuncture is hypothesized to work through metabolic remodeling, then the trial should be designed to test whether changes in metabolic markers partly mediate subsequent changes in cognition, mood, fatigue, dysphagia, or motor function. Without this step, the field will continue to accumulate associations without ever establishing whether observed metabolic shifts are causal, epiphenomenal, or merely correlative (62, 68, 70, 72).

Ultimately, the next generation of acupuncture-stroke research should look less like another broad efficacy trial and more like a staged translational program. Early studies should identify the most promising phenotype-biomarker pairs; mid-phase trials should test whether acupuncture can reproducibly shift those biomarker profiles under rigorous sham or active-comparator conditions; and later pragmatic studies should evaluate whether such biomarker-informed strategies improve long-term outcomes when added to standard rehabilitation and secondary prevention. Only by moving from undifferentiated efficacy claims to phenotype-specific, biomarker-anchored, mechanism-aware trials can the field determine whether acupuncture truly offers a meaningful metabolic contribution to stroke recovery rather than an inconsistent collection of context-dependent signals (67–73). Clinically, this framework should not be interpreted as positioning acupuncture as a substitute for reperfusion therapy, standard rehabilitation, or evidence-based secondary prevention. Rather, acupuncture should be evaluated as an adjunctive intervention that may have the greatest relevance in selected post-stroke phenotypes, particularly when conventional rehabilitation is combined with biomarker-guided monitoring and standardized outcome assessment.

## 9 Conclusion

Acupuncture in ischemic stroke should no longer be interpreted only through the traditional lens of local neuromodulation or nonspecific rehabilitation support. The more coherent reading of the current literature is that its potential effects may lie at the intersection of brain injury, systemic metabolic vulnerability, and phenotype-specific recovery biology. Clinical evidence suggests that acupuncture is not uniformly effective across all stroke outcomes, with the most consistent signals appearing in selected domains rather than in global disability as a whole. At the same time, mechanistic studies increasingly indicate that acupuncture may influence mitochondrial function, lactate handling, ferroptosis-related lipid injury, and microbiota-linked metabolic signaling, although these data remain predominantly preclinical. The central challenge for the field is therefore not simply to produce more trials, but to replace undifferentiated efficacy claims with biomarker-anchored, phenotype-specific, and methodologically rigorous studies that can determine which patients are most likely to benefit, at what stage, and through which biological pathways. Future research should therefore determine whether acupuncture has a defined adjunctive role in ischemic stroke recovery within rigorously designed, phenotype-specific, and biomarker-informed rehabilitation studies.
